# Bismuth Oxide Composite-Based Agricultural Waste for Wound Dressing Applications

**DOI:** 10.3390/molecules28155900

**Published:** 2023-08-05

**Authors:** Mayar Hassan, Mohamed A. Diab, Miral G. Abd El-Wahab, Abdelrahman H. Hegazi, Abdul-Hamid Emwas, Mariusz Jaremko, Mohamed Hagar

**Affiliations:** 1 Chemistry Department, Faculty of Science, Alexandria University, Alexandria 21321, Egypt; 2 National Research Center, Cellulose and Paper Department, 33El-Bohouth St. (Former El-Tahrir St.), Dokki, Giza 12622, Egypt; 3 Center of Excellence for Drug Preclinical Studies (CE-DPS), Pharmaceutical and Fermentation, Industries Development Centre (PFIDC), City of Scientific Research and Technological Applications (SRTA-City), New Borg El Arab 21934, Egypt; 4 Core Labs., King Abdullah University of Science and Technology, Thuwal 23955-6900, Saudi Arabia; 5 Smart-Health Initiative (SHI) and Red Sea Research Center (RSRC), Division of Biological and Environmental Sciences and Engineering (BESE), King Abdullah University of Science and Technology (KAUST), Thuwal 23955-6900, Saudi Arabia

**Keywords:** bismuth oxide composite, wound dressing, agriculture waste

## Abstract

The purpose of this study was to enhance the antimicrobial activity of bagasse paper by coating the paper with bismuth oxide (Bi_2_O_3_) and using it to accelerate the process of wound healing. Paper sheets were prepared from sugarcane waste (bagasse). First, the paper sheets were coated with different Bi_2_O_3_ concentrations to improve the antimicrobial activity of the paper. After that, the paper sheets were allowed to dry in an oven at 50 °C for 3 h. Then, in vitro antimicrobial activity was evaluated against different microbial species, including Gram-negative bacteria (i.e., Klebsiella pneumonia, Escherichia coli) and Gram-positive bacteria (i.e., Staphylococcus aureus, Streptococcus pyogenes). The obtained results showed that the paper coated with 25% and 100% Bi_2_O_3_ had activity against all models of bacteria; however, the paper coated with 100% Bi_2_O_3_ composite had the strongest inhibitory effect. Then, bagasse paper was coated with 100% Bi_2_O_3_ and different antibiotics, to investigate their wound-healing potency in a wounded rat model for 14 days. Moreover, the paper coated with 100% Bi_2_O_3_ inhibited the cellular migration in vitro. Conclusively, coating paper with Bi_2_O_3_ enhances the wound-healing potential when applied to wounds. This impact could be ascribed to Bi_2_O_3_’s broad antibacterial activity, which reduced infection and accelerated the healing process.

## 1. Introduction

Life on earth would not be possible in its current form if microbes and bacteria did not exist [1]. Microbes have an impact on our life in a variety of ways, including our health, food, agriculture, and environment. While some microbes are helpful, others are pathogenic or opportunistic [2,3]. According to the Center for Disease Control and Prevention, almost 3000 people die yearly from foodborne illnesses, including those caused by harmful bacteria [4]. Thus, the most effective strategy to prevent illnesses is to prevent bacteria from entering the body [5]. An infected person with persistent, difficult-to-heal wounds frequently experiences a cycle of pain, anxiety, diminished quality of life, and expensive treatment. The wound site must be protected with wound dressings to stop the spread of harmful bacteria and eliminate exudates. Hard-to-heal wounds are more prone to consequences such as infection, demanding more expensive interventions and more frequent dressing changes, which put more of a strain on the available resources. The dressing product needs to be reasonably priced and have a long shelf life for commercialization to succeed [6].

Hence, the rapid expansion of human and industrial activities leads to increased demand and overexploitation of natural resources, leading to grave environmental consequences. The pulp and paper industry can provide a good long-term solution for the problem of increased demand [7]. Recently, new technologies have used agricultural residues (wastes) to produce paper. This manufactured paper can be used for various purposes [8,9,10]. One of these alternative solutions is the introduction of non-wood fibers. Non-wood fiber refers to a group of plants with a wide variety of characteristics. Non-woody cellulosic plant materials such as straw, sugarcane bagasse, bamboo, cotton linter, and reeds are among the most frequently used non-wood fibers. Most non-wood plants mature quickly in a single growing season [11]. Bagasse can be present in all major paper categories, including packaging and boxes, printing, writing, photocopier paper, tissues, and newspaper. Chemical and mechanical pulping procedures are utilized to produce paper [12,13]. There is a worldwide tendency toward using paper instead of plastic. Paper in landfills decays or decomposes significantly faster than plastic. Plastic can take between 400 and 1000 years to degrade [14]. Discarded plastic that has made its way into the ground is acutely harmful and seriously destructive to agriculture. Thus, plastic should be restricted worldwide, and biodegradable alternatives would be a better solution for the toxicity problems of plastics [15].

Nanotechnology has attracted much interest in recent years, since it involves creating a material that is different from its source with better characteristics. One of the most important properties of the newly created materials is their large surface-area-to-volume ratio, which increases their capacity to penetrate cell membranes and assist in biochemical activities [16,17]. Recent advances in the approach and understanding of nanostructures have revealed their biological activity through changes in structure and functions produced in bacteria. New technologies have allowed better measurements of nanostructures’ size, shape, and surface chemistry, as well as their effects in terms of biocidal activities against microorganisms—bacterial and fungal [18,19]. Nanoparticles can enhance antimicrobial activity by serving as an excellent drug delivery system or exhibiting self-antimicrobial activity [20,21,22]. The efficient antimicrobial activity of nanoparticles is attributable to their impressive nano size and appreciable surface area [23,24]. The use of cellulose and bacterial cellulose [25] coupled with nanocomposites’ enhanced material properties has been investigated in various applications for disposable chemical sensors, energy conversion, biosensors [26,27,28], and antimicrobial activity [29,30]. 

Coating could be one of the processes that we can use to enhance material properties. Coatings can have many features, such as corrosion/wear resistance, increased surface hardness, altered surface texture, thermal/electrical insulation, and hydrophobicity [31]. To enhance the properties of paper filaments for wound healing, a nanocoating technique was used to add a bismuth substrate to the surface. Due to the hydrophilicity and porous nature of the fibers, and the consequent capillary forces, the filling of nanoparticles into paper molds is typically high [32,33]. Previous studies showed that coated nanoparticles have reduced biodegradability and can achieve therapeutic effects without systemic consequences [34]. In this research, we used Bi_2_O_3_ nanoparticles to coat paper filaments. Recently, Bi nanomaterials were found to be effective bacteriostatic materials, as they inhibit bacterial growth, while also showing low cellular toxicity [35,36]. It is believed that bismuth oxide nanocomposites may possess effective antimicrobial activity that could be useful to address pathogenic and resistant bacteria—especially in the case of wound-healing processes [37]. 

Wound dressings are made using a variety of techniques, according to their intended structure and material. Furthermore, various wound dressing structures have been explored in hopes of improving wound healing, including sponges [38], films [39], hydrocolloids [40], nanofiber membranes [41], and hydrogels [42].

The novelty of this work is the use of a paper sheet made of cellulose extracted from agricultural waste for wound healing. It is biodegradable, renewable, and biocompatible. Utilizing agricultural waste products as raw materials to create high-value-added materials benefits the environment and the economy.

This study used paper coated with bismuth oxide to accelerate wound healing. Bismuth oxide is known for its strong antimicrobial and anti-inflammatory activities [43], which can accelerate wound healing without any side effects. We applied bismuth oxide nanoparticles on a paper surface and investigated their effects on in vivo wound healing in a murine model and on in vitro antibacterial properties. It was demonstrated that the paper sheets coated with Bi_2_O_3_ were helpful in wound repair.

## 2. Result and Discussion

### 2.1. Fourier-Transform Infrared Radiation Spectroscopy

**Figure 1:**
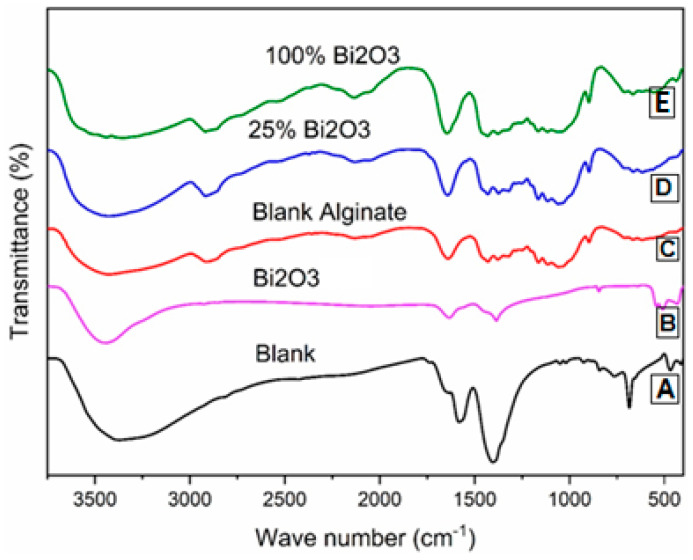
IR analysis: (**A**) blank; (**B**) Bi_2_O_3_; (**C**) blank alginate; (**D**) 25% Bi_2_O_3_; (**E**) 100% Bi_2_O_3_.

Fourier-transform infrared (FTIR) analysis (Figure 1) was carried out using FTIR spectroscopy at 400–4000 cm ^−1^. The FTIR spectra of Bi_2_O_3_ are shown in Figure 1B. According to Yuvakkumar and Hong [44], the wavenumbers 400–650 cm^−1^ and 3433 cm^−1^ correspond to BiO_6_ octahedron and -OH vibrations, respectively. The metal–oxygen (Bi-O) vibration causes the wide band at about 700–400 cm^−1^. The wide band at 2800–3200 cm^−1^ is caused by C-H stretching vibration and the -CH_2_ stretching at 2930 cm^−1^. The peak at 1386 cm^−1^ is typical of the NO_3_− group. The C=O, C-N, and C-C stretching are assigned to the wavenumbers of 1622, 1401, and 1076 cm^−1^, respectively [45,46]. In the case of the blank (Figure 1A), the presence of C-H and -OH functional group moieties was clearly seen in cellulose. The broad absorption bands at around 3500 cm^−1^ and 2920 cm^–1^ were related to the presence of the OH group of cellulose and C-H stretching vibrations, respectively. The peak for C-H and C-O vibrations contained in the polysaccharide rings of cellulose was in the range of 1400–1650 cm^−1^ [47,48]. In the case of 25% Bi_2_O_3_ (Figure 1D) and 100% Bi_2_O_3_ (Figure 1E), the broad bands at 3500 cm^−1^ became weak because the addition of Bi_2_O_3_ could decrease the intensity of the OH peak. However, it is well understood that the infrared spectrum is congested with bands that overlap between cellulose and Bi_2_O_3_. A decrease in the broad bands at 3500 cm^−1^ could confirm that Bi_2_O_3_ was loaded successfully on the cellulose surface of the paper sheets, but there was overlapping of the cellulose and Bi_2_O_3_ peaks. In 25% Bi_2_O_3_ and 100% Bi_2_O_3_, subtle differences in the spectra could also be seen in the range between 1400 and 1650 cm^−1^ due to overlap between the cellulose and Bi_2_O_3_ peaks. In the case of blank alginate (Figure 1C) the peaks appeared much less sharply than for the blank, as a result of the interaction of the coated material with cellulose, which reduces the possibility of the clear appearance of cellulose. Interestingly, as the proportion of Bi_2_O_3_ coated on the paper surface increased, the intensity of the absorption peaks of Bi_2_O_3_ was enhanced. This elucidates the formation of convenient interactions between the coating layer and paper sheet bands. 

### 2.2. X-ray Diffraction Analysis

**Figure 2:**
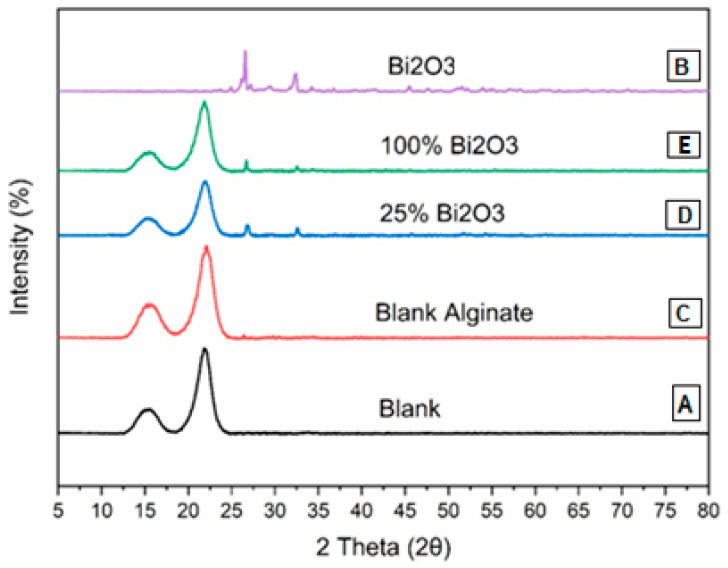
XRD of (**A**) blank, (**B**) Bi_2_O_3_, (**C**) blank alginate, (**D**) 25% Bi_2_O_3_, and (**E**) 100% Bi_2_O_3_.

The XRD patterns of the (A) blank, (B) Bi_2_O_3_, (C) blank alginate, (D) 25% Bi_2_O_3_, and (E) 100% Bi_2_O_3_ are presented in Figure 2. Figure 2B shows the XRD pattern of Bi_2_O_3_, with a strong and sharp peak, showing that the Bi_2_O_3_ is in the crystal phase. The peaks correspond to the monoclinic crystal phase of Bi_2_O_3_ at the 2θ values of 24.55°, 25.75°, 26.92°, 27.34°, 33.18°, 35.00°, 35.42°, and 37.56°. These samples exhibited peaks around 2θ = 14.5° and 22.5°. These are meant to approximate the usual cellulose structure [49]. Meanwhile, the intensities of the typical cellulose peaks were lower in 25% Bi_2_O_3_ (Figure 2D) and 100% Bi_2_O_3_ (Figure 2E), due to the long peaks of cellulose in the blank (Figure 2A), which blended to form shorter peaks of Bi_2_O_3_ (Figure 2B). The XRD pattern of Bi_2_O_3_ (Figure 2B) shows sharp peaks around the 2θ values of 25.75°, 27.34°, and 33.5° [44], which appear at lower intensity in 25% Bi_2_O_3_ (Figure 2D) and 100% Bi_2_O_3_ (Figure 2E). The XRD patterns show that there is an interaction between the Bi_2_O_3_ and cellulose bands in the cases of 100% and 25% Bi_2_O_3_, as their pattern matches that in cellulose and in Bi_2_O_3_ nanoparticles.

### 2.3. Scanning Electron Microscopy (SEM)

**Figure 3:**
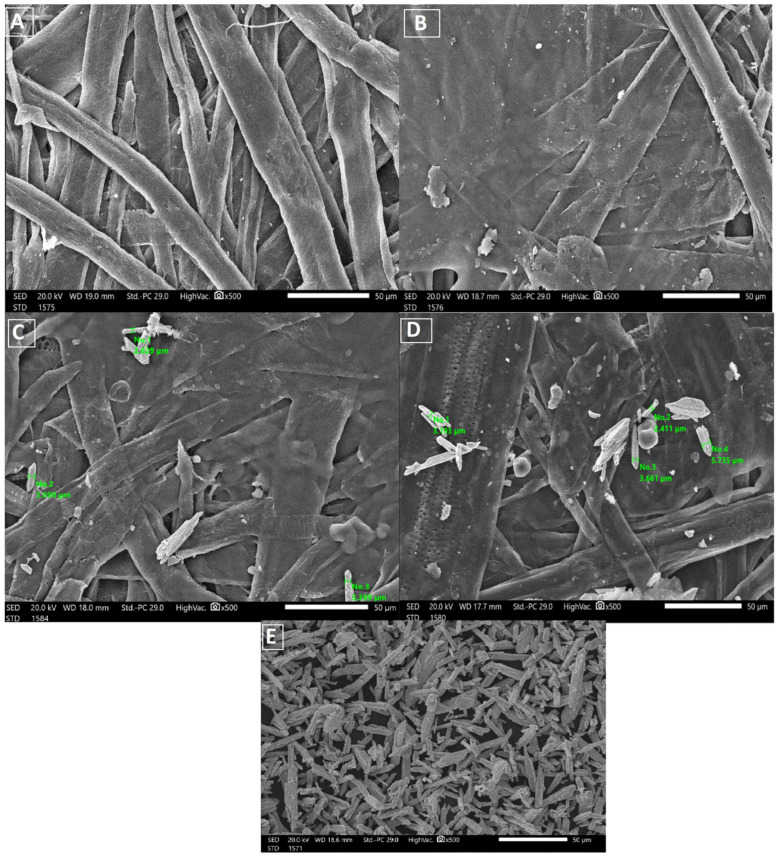
SEM of (**A**) blank, (**B**) blank alginate, (**C**) 25% Bi_2_O_3_, (**D**) 100% Bi_2_O_3_, and (**E**) Bi_2_O_3_.

The structure of the uncoated and coated paper sheets was investigated by SEM. The SEM images of the uncoated and coated paper sheets are shown in Figure 3. It was obvious from the comparison of photograph (a) with (b), (c), and (d) that the blank paper sheets prepared from bleached bagasse pulp and coated sheets appeared smooth, with no pores, and more homogeneous than the untreated sheets (blank). The SEM images show the deposition of Bi_2_O_3_ on the surface of the coated paper. Based on the SEM observations, it should be noted that the coated particles were much more abundant in the case of 100% Bi_2_O_3_ than in 25% Bi_2_O_3_. The images also show the size of the Bi_2_O_3_-coated paper sheets. Figure 3E shows the bismuth nanoparticles. The SEM images show that the Bi_2_O_3_ NPs were distributed on the paper surface in different proportions, confirming the existence of Bi_2_O_3_ nanoparticles. 

### 2.4. Mechanical Properties

Various mechanical properties of the paper were evaluated, including the bulk density, maximum load, breaking length, elongation at maximum load, tensile index, stiffness, and young’s modulus of the dried samples (Table 1). Modification of the sugarcane bagasse paper with Bi_2_O_3_ presented higher breaking length results than without modification. The results indicated that the paper coated with 25% Bi_2_O_3_ and 100% Bi_2_O_3_ had better mechanical features compared with the other coated papers.

**Figure 4:**
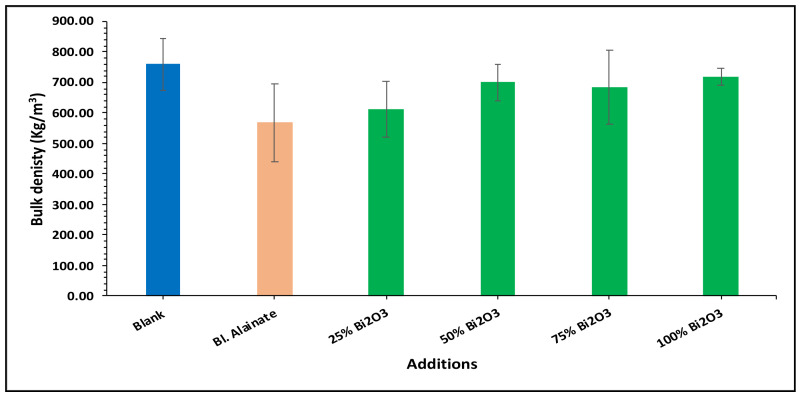
Bulk density of blank, blank alginate, 25% Bi_2_O_3_, 50% Bi_2_O_3_, 75% Bi_2_O_3_, and 100% Bi_2_O_3_.

**Figure 5:**
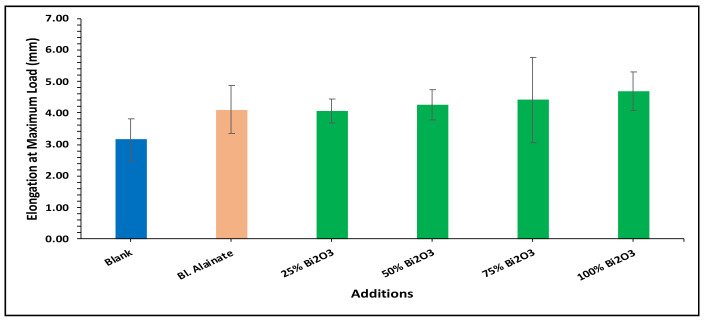
Elongation at maximum load of blank, blank alginate, 25% Bi_2_O_3_, 50% Bi_2_O_3_, 75% Bi_2_O_3_, and 100% Bi_2_O_3_.

**Figure 6:**
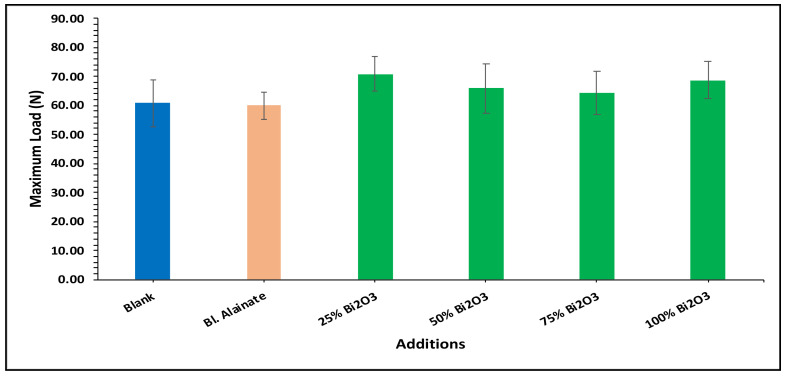
Maximum load of blank, blank alginate, 25% Bi_2_O_3_, 50% Bi_2_O_3_, 75% Bi_2_O_3_, and 100% Bi_2_O_3_.

**Figure 7:**
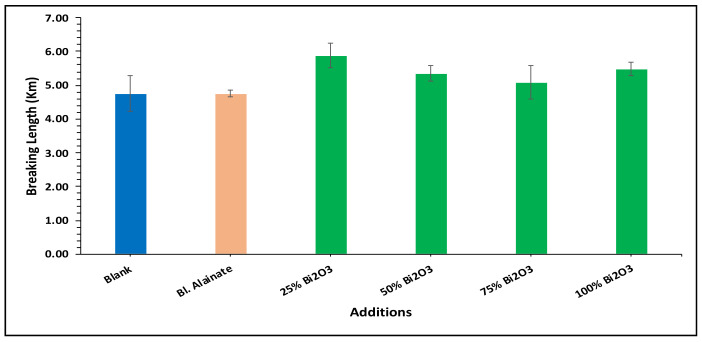
Breaking length of blank, blank alginate, 25% Bi_2_O_3_, 50% Bi_2_O_3_, 75% Bi_2_O_3_, and 100% Bi_2_O_3_.

**Figure 8:**
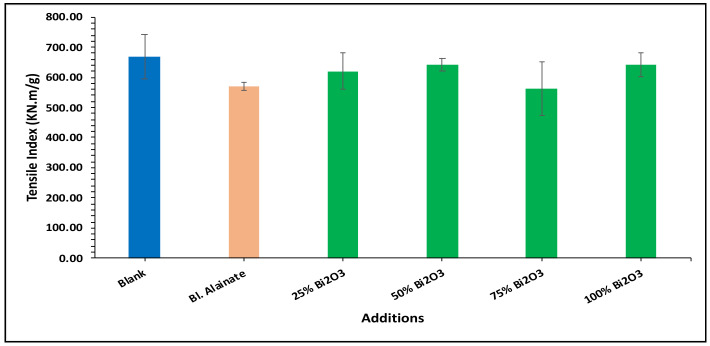
Tensile index of blank, blank alginate, 25% Bi_2_O_3_, 50% Bi_2_O_3_, 75% Bi_2_O_3_, and 100% Bi_2_O_3_.

**Figure 9:**
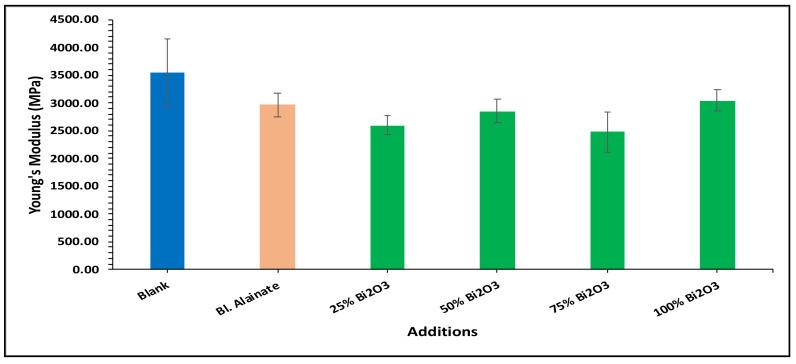
Young’s modulus of blank, blank alginate, 25% Bi_2_O_3_, 50% Bi_2_O_3_, 75% Bi_2_O_3_, and 100% Bi_2_O_3_.

In the bulk density results presented in Figure 4, the blank has the greatest bulk density, followed by 100% Bi_2_O_3_, while in Figure 5 the 100% Bi_2_O_3_ and 75% Bi_2_O_3_ show higher percentage values for elongation at maximum load in comparison to the blank. In Figure 6 and Figure 7, 25% Bi_2_O_3_ shows higher values of maximum load and slightly lower breaking length than 100% Bi_2_O_3_, which appears at roughly the same level. In Figure 8, the paper sheets’ tensile strength shows higher values in 50% Bi_2_O_3_ and 100% Bi_2_O_3_. In Figure 9, the Young’s modulus indicates a specimen’s stiffness; it was enhanced by increasing the percentage of Bi_2_O_3_, with 100% Bi_2_O_3_ showing increased stiffness compared to the other coated paper samples [45,46].

### 2.5. Antimicrobial Activity

**Figure 10:**
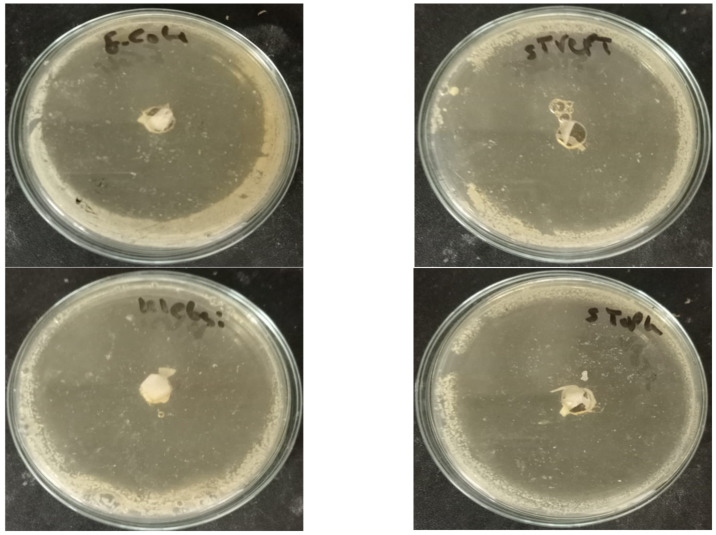
Optical images of the inhibition zones of paper coated with 100% Bi_2_O_3_.

**Figure 11:**
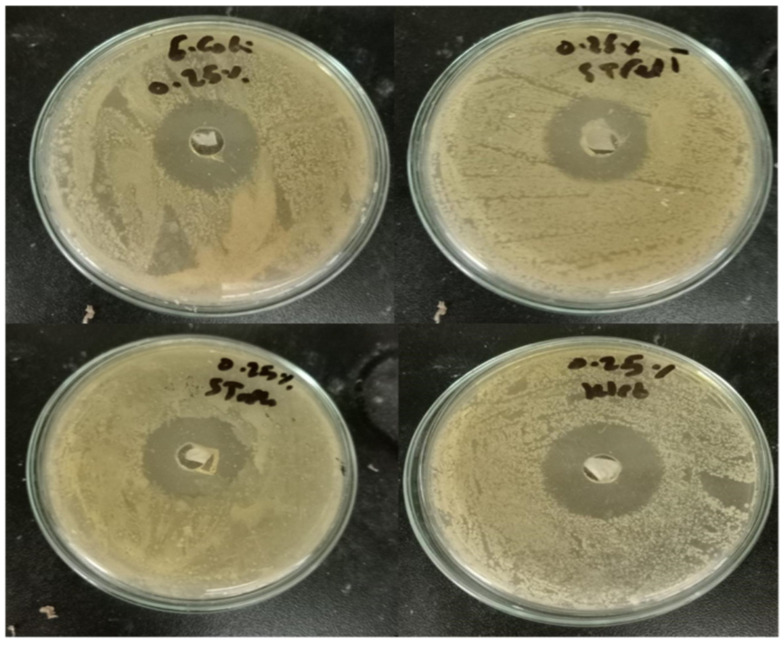
Optical images of the inhibition zones of paper coated with 25% Bi_2_O_3_.

The disk diffusion technique was used to evaluate the antimicrobial activity of the prepared paper sheet. As shown in Table 2 and Figure 10 and Figure 11, the 25% Bi_2_O_3_ and 100% Bi_2_O_3_ showed antimicrobial activity against all of the model pathogens applied, raising the percentage of bismuth-enhanced antimicrobial activity against all of the model microbes. These results indicated that increasing the percentage of bismuth was correlated with increased antibacterial activity. Based on the diameters of the inhibition zones and the results shown in Table 2, the paper coated with 100% Bi_2_O_3_ had the highest antimicrobial activity.

The individual physical and chemical properties of Bi_2_O_3_ outperformed those of traditional organic and synthesized antimicrobial agents, showing smaller crystal sizes, smaller average particle size, greater stability, and a greater ability to interact with more pathogenic bacteria and *Candida* species, boosting their antimicrobial potential [50].

### 2.6. Healing Test Method

**Figure 12:**
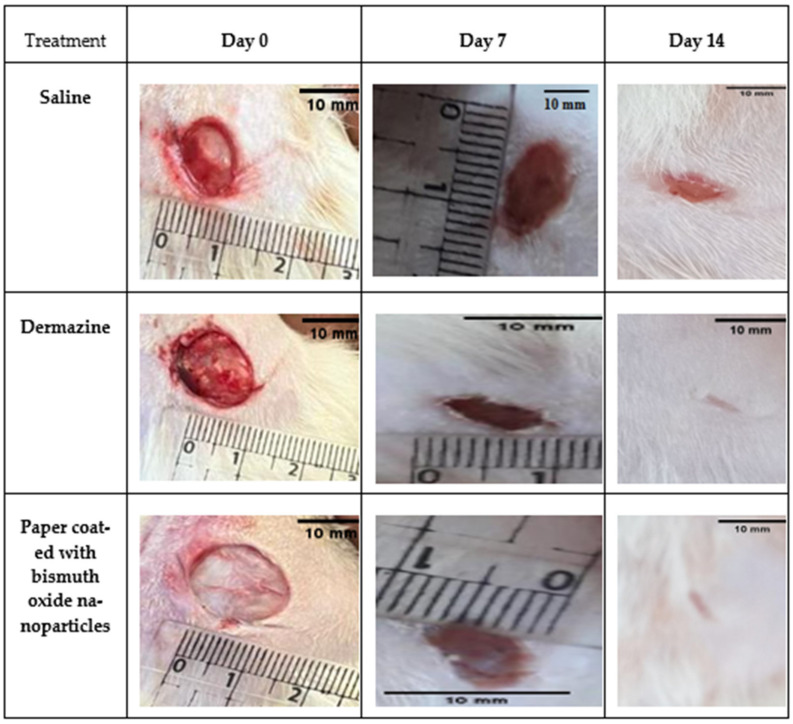
Digital images of macroscopic wound size and condition compared with the initial value at day 0.

Figure 12 shows that in comparison to saline, traditional dressings, and dermazine groups, within 7–14 days after an injury, paper coated with bismuth oxide demonstrated effective wound healing and encouraged quicker and better reepithelization. It was also permeable and left no residue on the skin. In the early post-injury days, exudate could not be seen in the wounds. At day 7, all wounds shrank in proportion to the day of injury, but differences could be seen when compared to the dermazine and saline groups. A crust appeared all over the wound when using paper coated with bismuth oxide, indicating that the wound had dried. The wound was uncontaminated. No exudate, inflammation, or microbial contamination (e.g., pus or edema) was recorded at day 14; the wounds reduced in size, and the dermazine and paper coated with bismuth oxide groups both exhibited a full reepithelization. In comparison to the dermazine group and the control group, the results showed that paper coated with bismuth oxide promoted faster wound healing. It was also simple to apply as a wound dressing and had good adhesion to the wound bed.

**Figure 13:**
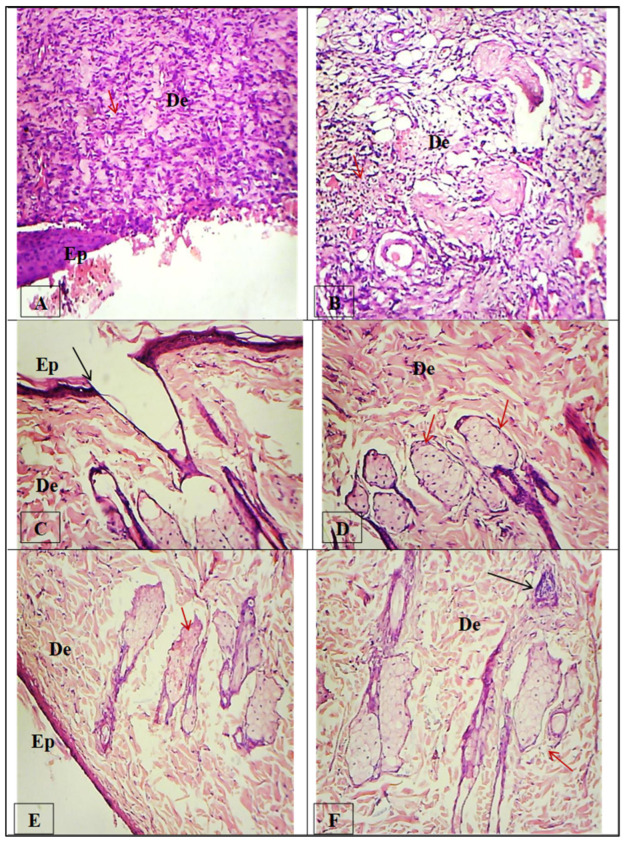
Histological evaluation of skin wound healing at day 14.

Photomicrographs of sections of skin tissues in Figure 13 showing H&E staining. (A,B) Injured rat skin treated with control saline, showing a thick migration tongue of the epithelium, with the wound not completely closed at the center; increased inflammatory cells (Ep) (Red arrow) and inner layer dermis (De) (layer of connective tissue), with no sebaceous glands or hair follicles but inflammatory cells still present (Red arrow). (C,D) Injured rat skin treated with a market drug (dermazine), showing the wound almost completely sealed by reepithelization (EP) over the dermis (De) cell layer, with a small area at the wound’s center that is still not fully formed (Black arrow); no infiltration of inflammatory cells was found, and the sebaceous glands were fully developed (Red arrows). (E,F) Injured rat skin treated with paper coated with bismuth oxide, showing the wound completely healed and sealed by thin reepithelization (EP) over the dermis (De) cell layer; infiltration of inflammatory cells was found at the epidermis layer (Black arrow), and the sebaceous glands were fully developed (Red arrows).

Table 3 displays the percentage of wound contraction for the various treatment groups at days 0, 7, and 14. These findings suggest that the paper coated with bismuth oxide demonstrated strong wound-healing potential. This may be explained by the broad antimicrobial activity of bismuth oxide, which reduces the infection and speeds up the healing.

### 2.7. In Vitro Cell Migration

**Figure 14:**
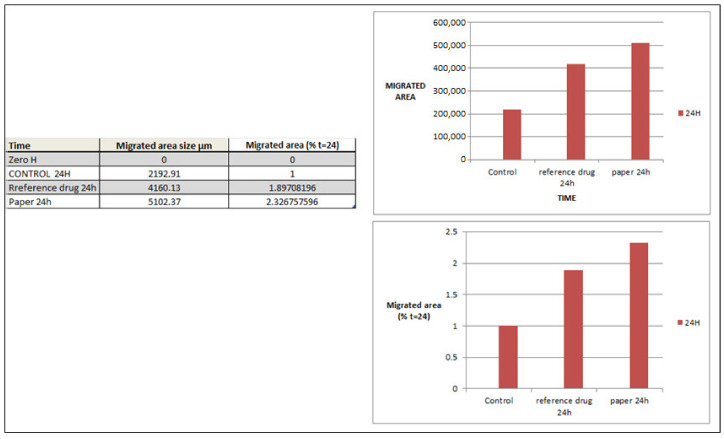
The graphical data of migration area determined by cell migration assay.

The wound-healing test is a simple and useful technique for assessing cell migration ability, where the cells are pretreated with drugs before the scratch-making experiment begins. It may be possible to determine whether or not cell migration occurs by observation. The cells surrounding the scratch grow and migrate forward to the center of the injured area. Similarly, three other experiments were conducted for comparison, in which cells were individually incubated with the control, reference drug (dermazine), and paper before being scratched. After being treated for several hours in the three different experiment groups, it was discovered that cells clearly migrated to the injured area. Scratch distances and width closure were calculated from time 0 to 24 h. Figure 14 shows that a significant increase in the number of migratory cells was observed. After being treated with the reference drug and paper, but at a higher rate in the case of paper, we found synergistic effects, with increased cell migration and invasion compared with that in untreated cells. These results indicate that the paper enhanced the migration of cells and accelerated the wound healing. It has been reported by Soliman et al. [51] in an in vitro study that cellulose nanocomposites enhanced endothelial cell migration; however, Silva et al. [52] found that nanofibrous nanocellulose patches showed a high migration capacity, resulting in nearly complete occlusion of the wound. The present study investigated paper sheets coated with Bi_2_O_3_ nanoparticles that promoted healing, which could be attributed to antimicrobial activity that helps prevent infection and skin damage. Among the greatest benefits of our research are the environmental advantages and low cost of the sheets prepared from agricultural waste.

## 3. Materials and Methods

The bagasse fibers were supplied by Quena Paper Industry Co., Egypt. The bagasse raw material and bleached kraft bagasse pulp were analyzed according to TAPPI standard methods—namely, α-cellulose 41.50, 73.70% (T-203 cm-99); lignin 20.40, 1.18% (T-222 om-88); and pentosane 27.20, 24.40% (T-223 cm-84). Sodium alginate (SA) (food grade) was purchased from LOBA Chemie. Bi(NO_3_)_3_·5H_2_O (99% in purity), HNO_3_ (70% in purity), citric acid, and Tween 80 were bought from Sigma-Aldrich and used exactly as they were given. Data are presented as the mean, and all measurements were carried out in triplicate.

### 3.1. Synthesis of Bismuth Oxide

Bi_2_O_3_ was synthesized using bismuth nitrate and citric acid. At a 1:1 molar ratio, equimolar quantities of Bi(NO_3_)_3_·5H_2_O and citric acid were dissolved in nitric acid solution and mixed well. A small quantity of Tween 80 was added as a surfactant to prevent agglomeration. The pH of the solution was raised to 3. After stirring the aforementioned solution for 2 h, a sol developed. For 3 h, the sol solution was heated to 80 °C to create a yellowish gel. This gel was heated in an oven at 120 °C. The gel began to inflate and fill the beaker, forming a frothy forerunner. This foam was made up of homogeneous flakes with very small particles.

### 3.2. Hand-Sheet Making

To make paper sheets out of sugarcane waste (bagasse), the bagasse was subjected to two chemical treatments: firstly, soda kraft pulping to remove the lignin using 15% NaOH with 12% sulfidity based on the oven-dried raw material at a 1:3.5 liquor ratio for 30 min at 160–170 °C and 6 bar pressure, followed by a bleaching process consisting of three stages using chlorine dioxide, oxygen, and chlorine dioxide to isolate bleached cellulose pulp (according to the conditions applied by Quena Paper Industry Co., Cairo, Egypt). According to the S.C.A standard (S.C.A model-AB Lorentzen and Wettre), traditional hand-sheets were produced using a sheet-former with a base weight of 80 g/m^2^. About 1.8 g of pulp was cut off for each sheet, and then 5–7 L of water was added to the pulp slurry. After a stirring and agitating step, the suspension was separated by suction through a screen. In the appliance, a sheet with a surface area of 226.98 cm^2^ and a diameter of 170 mm was formed and then pressed for 4 min with a hydraulic press. The wet sheet was then collected on blotting paper, located between two sheets. The sheets were dried in a rotary drum dryer for 4 h at 105 °C. Tensile strength testing was performed with a universal testing machine (LR10K; Lloyd Instruments, Fareham, UK) equipped with a 100 N load cell and a constant crosshead speed of 2.5 cm/min, following the TAPPI (T494-06) standard method. The gauge length was 100 mm, and 15 mm wide strips were used for the analysis.

### 3.3. Preparation of Coating Solutions

A solution of 25 mL of (2% *w*/*v*) alginate with 0.5 g of bismuth oxide was prepared and diluted to various proportions with (2% *w*/*v*) alginate (25%, 50%, 75%, and 100%), and then applied on the surface of the paper using a 120-micron coating applicator (a type of film applicator combining 4 gap sizes in one unit i.e., 30, 60, 90, and 120 μm). The coated sheets were dried in a 50 °C oven for 3 h.

### 3.4. Characterization of Papers

#### 3.4.1. Fourier-Transform Infrared (FTIR) Spectroscopy

Measurements were taken on an FTIR Bruker Tensor 37 (Bruker, Billerica, MA, USA) in the 400 to 4000 cm^−1^ wavenumber range (spectral resolution 2 cm^−1^). The samples were prepared as thin films formed by pressing a solid–solid mixture of 1% sample and 99% spectral-grade KBr; the paper sheet sample was placed on top of an integrating sphere, and one spectrum was acquired from each face (transversal, tangential, and radial), yielding six distinct spectra for each sample, for a total of eight per species.

#### 3.4.2. X-ray Diffraction Analysis

We examined the structure of the samples by X-ray powder diffraction (XRD, D2 PHASER, Bruker, Billerica, MA, USA). The diffraction angle (2θ) was changed from 0 to 100°. Monochromatic CuKα radiation (l = 1.54 Å) was used to determine the structure.

#### 3.4.3. Scanning Electron Microscopy

The morphology of the samples was analyzed using SEM (JEOL-JSM-IT200, Tokyo, Japan).

#### 3.4.4. Mechanical Properties

Tensile strength testing was performed with a universal testing machine (LR10K; Lloyd Instruments, Fareham, UK) equipped with a 100 N load cell and a constant crosshead speed of 2.5 cm/min, in accordance with the TAPPI (T494-06) standard procedure. The gauge length was set to 10 cm, and 15 mm wide strips were utilized for the analysis.

### 3.5. Antimicrobial Activity

Antimicrobial activity was investigated by agar well diffusion assay [53] and the ASTM E2149-01 standard test method for detecting the antimicrobial activity of antimicrobial agents under conditions of dynamic contact for all samples. We investigated four microbial species known to be pathogenic, including Gram-negative bacteria (Klebsiella pneumonia ATCC700603, Escherichia coli ATCC25922) and Gram-positive bacteria (Staphylococcus aureus ATCC25923 and Streptococcus pyogenes EMCC1772). For 24 h, the bacteria were cultured in nutrient broth at 37 °C. Paper sheet samples (2 g) were cut into small pieces (1 × 1 cm) and transferred to a 250 mL Erlenmeyer flask containing 50 mL of the working bacterial dilution. All flasks were loosely capped, placed on the incubator, and shaken for 1 h at 37 °C and 120 rpm with a Wrist Action incubator shaker. Then, 100 μL of each inoculum (1 × 10^8^ cfu/mL) was inculcated in agar media and placed on a Petri plate. All of the tested bacteria were cultured at 37 °C for 24 h. The diameter of the inhibition zone encircling the well (mm), including the well diameter, was used to calculate the zone of inhibition. The readings were obtained in triplicate in three different fixed directions, and the average results were recorded.

### 3.6. Healing Test Method

#### 3.6.1. Full-Thickness Wound Model Preparation

Adult male Sprague Dawley male rats weighing 180 to 200 g were used in the wound-healing paradigm. All animal groups were housed in their own stainless steel cage with controlled light and room temperature (25 3 °C; 35–60% humidity) on a 12 h light/dark schedule. The rats were given a standard rat diet of pellets and water as needed [54].

The Local Ethics Committee on Animal Research authorized the research (approval number AU04220924102). The animals were weighed, numbered, and divided into three groups of five each: Group I served as a vehicle control and received saline. Group II served as a control group and received ointment (dermazine); this product is made up of two components: iodoquinoline antibiotics, and hydrocortisone—a moderate corticosteroid that acts as an anti-inflammatory. Finally, Group III was treated with paper coated with bismuth oxide. For surgical procedures, the rats should not have had any preexisting skin lesions at the surgery site. The animals were sedated with 10% ketamine hydrochloride (Dopalen^®^, 0.1 mL/100 g body weight) and 2% xylazine hydrochloride (Calmium^®^, 0.1 mL/100 g body weight) intramuscularly before being shaved on the back. Following shaving, the region was antiseptically treated with 4% alcohol-based iodine. Using sterile surgical scissors, a circular (diameter 1.5 cm) and surgical full-thickness open excision wound was created to the depth of the loose subcutaneous tissue on the upper back on both sides of the shaved area [55]. All animal groups were housed in their cages for 14 days, and the wounds were cleansed daily with alcohol before a determined dose of therapy was applied topically to each group. During the experiment, the wound was wrapped with sterile gauze and held in place with circular adhesive bands [56].

#### 3.6.2. Preliminary Analysis of Wound Closure Rates

On postoperative days 0–14, the experimental animals were shot against the backdrop of a metric ruler with a digital camera. The limits of grossly visible epithelialization were used to define the wound closure area, with all surface areas in a two-dimensional plane calibrated against the neighboring metric ruler. Each mouse had four separate photomicrographic measurements taken. The wound-healing percentage (% contraction) was computed as follows:

(1) $$\%\;\mathrm{Wound}\;\mathrm{Contraction}=\frac{HealedArea}{TotalArea}$$

#### 3.6.3. Histopathological Analysis

Following scarification, skin samples were taken from all groups of rats and preserved in 10% neutral buffered formalin solution. After at least 24 h, dehydration in increasing degrees of ethanol, clearing in xylene, and embedding in paraffin wax were performed. Tissue slices (3–5 microns thick) were cut and stained with hematoxylin and eosin (H&E) as described by Bancroft et al. (1996) [57], and histopathological examination was performed using light microscopy (×400).

### 3.7. In Vitro Cell Migration

The wound-healing assay was used to determine migration [58]. In a 12-well dish, cells were seeded and incubated until confluence. To produce a cell-free wound area, the cells were scratched with a pipette tip. The cells were then treated with a double sample of Bi_2_O_3_-coated filter paper and a double sample of antibiotic-treated filter paper (Dermazine). Half of the wells were covered by the paper sheet. The dish was raised to a 45-degree angle on the first day to enable the cells to settle on the sheet, and then returned to its horizontal position the next day to observe cell migration for 24 h.

## 4. Conclusions

In this work, bagasse paper coated with Bi_2_O_3_ was produced. The presence of nanoparticles on the paper’s surface was confirmed by FTIR; the results showed an interface between the cellulose bands and the Bi_2_O_3_ nanoparticles bands. The XRD peaks also showed the presence of Bi_2_O_3_ nanoparticles. The SEM results proved the deposition of nanoparticles on the paper surface. The mechanical properties showed the best results for paper sheets prepared from bleached bagasse coated with 25% and 100% Bi_2_O_3_ compared with the other coated paper sheets. The antimicrobial activities of the paper with the best mechanical properties demonstrated a strong inhibitory effect against all model bacteria tested. However, the paper coated with 100% Bi_2_O_3_ showed the greatest efficacy. The biological activity investigation of the paper coated with 100% Bi_2_O_3_ showed enhanced wound healing, and the tissue regeneration activity in the wounded rat model was investigated at day 7 and day 14. On day 7 the wound closed by 81.86% for the coated paper, 76.31% for dermazine, and 14.21% for saline; these results prove the ability of the paper sheets to accelerate the healing of the wounds. For the in vitro cell migration model, the coated paper sheets showed the highest efficiency for cell migration. Furthermore, the bismuth oxide nanoparticles’ composition serves to control infection by preventing microorganisms’ growth, as it has an antimicrobial effect. It is suggested that the paper coated with 100% Bi_2_O_3_ could be regarded as a possible dressing material after further clinical evaluations.

## Figures and Tables

**Table 1:** Mechanical properties of blank, blank alginate, 25% Bi_2_O_3_, 50% Bi_2_O_3_, 75% Bi_2_O_3_, and 100% Bi_2_O_3_.

Sample ID	Bulk Density (Kg/m^3^)		Maximum Load (N)		Breaking Length (m)		Elongation at Maximum Load (mm)		Stiffness (N/m)		Tensile Index (N·m/g)		Young’s Modulus (MPa)	
Blank	759.59		60.78		4.75		3.15		58.50		669.92		3545.17	
Blank alginate	568.59	−25.15	59.91	−1.43	4.75	0.02	4.10	29.98	48.88	−16.43	570.12	−14.90	2962.67	−16.43
25% Bi_2_O_3_	612.54	−19.36	70.87	16.59	5.87	23.67	4.07	29.21	50.66	−13.40	619.32	−7.55	2597.78	−26.72
50% Bi_2_O_3_	699.80	−7.87	65.84	8.32	5.35	12.64	4.26	35.08	53.51	−8.52	641.53	−4.24	2854.04	−19.50
75% Bi_2_O_3_	684.37	−9.90	64.33	5.83	5.08	7.03	4.41	39.88	48.26	−17.50	562.32	−16.06	2474.69	−30.20
100% Bi_2_O_3_	718.31	−5.43	68.78	13.16	5.48	15.36	4.70	48.92	54.73	−6.43	642.98	−4.02	3040.80	−14.23

**Table 2:** Antimicrobial activity of paper coated with 25% Bi_2_O_3_ and 100% Bi_2_O_3_.

Pathogenic Strain	Inhibition Zone Diameter (mm) **
Gram-negative bacteria	
Klebsiella pneumonia ATCC700603	
Paper 25%	30
Paper 100%	71
Antibiotic (0.1%)	38
Escherichia coli ATCC25922	
Paper 25%	27
Paper 100%	67
Antibiotic (0.1%)	40
Gram-positive bacteria	
Staphylococcus aureus ATCC25923	
Paper 25%	29
Paper 100%	68
Antibiotic (0.1%)	30
Streptococcus pyogenes EMCC1772	
Paper 25%	25
Paper 100%	69
Antibiotic (0.1%)	28

** Inhibition zone diameters include the 5 mm well diameter.

**Table 3:** Percentage of wound contraction of the different treatment groups.

Days	Saline		Dermazine		Paper Coated with Bismuth Oxide	
	Area (cm^2^)	Closure %	Area (cm^2^)	Closure %	Area (cm^2^)	Closure %
0	9.39 ± 0.57	Nil	13.96 ± 0.77	Nil	19.07 ± 0.05	Nil
7	8.06 ± 0.62	14.21	3.31 ± 0.45	76.31	3.46 ± 0.15	81.86
14	5.80 ± 0.08	38.80	0.00 ± 0.00	100	0.00 ± 0.00	100

## Data Availability

All of the original data are available upon reasonable request from the corresponding authors.

## Sample Availability

Samples of the compounds are not available from the authors.
